# The impact of health and environmental messaging with and without product filtering in complex retail markets: the case of pulses

**DOI:** 10.3389/fnut.2024.1454271

**Published:** 2024-09-25

**Authors:** Christopher R. Gustafson, Henriette Gitungwa, Sushil C. Sapkota, Devin J. Rose

**Affiliations:** ^1^Department of Agricultural Economics, University of Nebraska-Lincoln, Lincoln, NE, United States; ^2^Department of Food Science and Technology, University of Nebraska-Lincoln, Lincoln, NE, United States; ^3^Department of Agronomy and Horticulture, University of Nebraska-Lincoln, Lincoln, NE, United States; ^4^Nebraska Food for Health Center, University of Nebraska-Lincoln, Lincoln, NE, United States

**Keywords:** food choice, search costs, pulses, nutrition, sustainability, environment, filtering, messaging

## Abstract

**Introduction:**

Multiple barriers exist to healthy and environmentally sustainable food choices. Limited consumer understanding of the health and environmental implications of food choices complicates their abilities to make choices that lead to desired outcomes. The complexity of the retail environment itself may crowd out less immediate motivations to address health or environment. Even if consumers understand general impacts of food choices on health and environmental outcomes, there may be non-negligible time and search costs to identifying the products that meet consumers’ needs. In many food categories, the foods containing attributes that help achieve health and sustainability outcomes may represent a small percentage of available products. In this research, we examine the case of pulses—beans, chickpeas, lentils, and dry peas. Pulses are nutritious and have a low environmental impact. However, consumption of pulses in the US is quite low, which may be attributable to low consumer knowledge of pulse benefits, as well as difficulty of identifying pulse products in retail environments.

**Methods:**

In this research, we examine the choice of pulse-based foods in three conditions: (1) a control condition, (2) a messaging condition communicating the health and environmental benefits of pulse products, and (3) a paired messaging condition with a choice environment intervention that allows respondents to choose to filter products to those that contain pulses. Participants selected a food item from each of six food categories.

**Results:**

We find slight, but significant, increases in pulse choice in the messaging only condition relative to the control condition, but dramatic and highly significant increases when participants can filter the products to easily view pulse products. We also find evidence for knowledge being a barrier to healthy/sustainable food choice. Participants exposed to the messaging were more likely to view pulses as environmentally beneficial, and less likely to report that they did not know the health or environmental impacts of pulse foods.

**Discussion:**

We find that paired messaging and filtering interventions significantly increase the choice of pulse-based foods, which offer both human health and sustainability benefits.

## 1 Introduction

The modern food system has multiple concerning impacts on society. Poor diet, linked to rising consumption of ultra-processed foods, contributes to high rates of overweight and obesity (1), and is a leading cause of morbidity and mortality in the US and globally (2, 3). High intake of ultra-processed, calorie-dense foods and low intake of nutrient-dense, antioxidant-rich foods, such as pulses, whole grains, and fruits and vegetables, leads to weight gain (4), which is linked to increased risk of type-2 diabetes, cancer, and heart disease (5). The economic burden of poor diet has been estimated to annually cost the US $150 billion in direct costs and $3–6 billion in indirect costs (6).

At the same time, increasing concerns about the environmental impacts of food production systems has led to greater scrutiny of the impact of food production on the environment and climate (7). While livestock production has been identified as a high emitter of greenhouse gases (GHGs)—producing approximately 15% of anthropogenic GHGs (8), the production of plant-based foods has a much smaller environmental impact. A recent study found that meat-containing meals had an environmental impact that was 14 times higher than vegan versions of those meals, while vegetarian versions of the meals were 3 times more impactful than vegan versions (9). Pulses—such as beans, chickpeas, lentils, and dry peas—are a good source of protein, and so are frequently used as plant-based substitutes for animal-based proteins (10) in order to maintain nutrient profiles while reducing environmental impacts. A study estimating the effect of substituting pulses for beef found that this change alone would achieve 50–75% of the US’s target reduction of GHGs (11). Research on acceptability of alternative proteins found that plant-based proteins—such as those found in pulses—are a more acceptable substitute to consumers for conventionally produced animal-source proteins than other alternative protein sources, such as insects and lab-grown meat (12). Pulses also offer an array of other beneficial nutrients, such as dietary fiber (13), which was identified by the Dietary Guidelines for Americans 2020–2025 as an underconsumed nutrient of public health concern (14). Thus, interventions designed to increase consumer demand for food products containing pulses may be an important pathway to improving the nutritional quality of diets and mitigating climate impacts of food production.

Pulses are healthy (13, 15), environmentally sustainable (16), and economical. Many consumers report basing food decisions on health, environment, and cost attributes (17–19), though different segments of consumers may place different relative values on these outcomes (20). Despite offering numerous, diverse benefits, consumption of pulses in the U.S. is significantly below recommended levels and, in fact, has been decreasing in recent years (21, 22). Barriers to pulse consumption include a lack of knowledge about/belief in the benefits that pulses offer; perceived negative impacts, such as flatulence; and a relatively small footprint of pulse-based products, defined as products with a pulse as one of the first three ingredients, in a crowded retail environment (23–26). Pulse-based products represented only 4% of branded food products in the US (27). Variation in awareness of pulses may mean that consumers are unfamiliar with the diversity of pulse types and the effects of consuming different pulses. For instance, beans are likely familiar to most Americans, while lentils and chickpeas may be less well known. However, research shows that a common barrier to consumption—flatulence (24, 28)—does not result from the consumption of a large number of pulses, including lentils, chickpeas, and green peas (29).

A significant amount of experimental research has been devoted to identifying effective ways to motivate changes in consumer behavior to both improve human health and increase the sustainability of food production systems by shifting consumer purchases toward more sustainable products (30–37). However, much of this research uses simple choice sets, typically featuring two to four items at a time, to test labeling or informational strategies to promote healthier or more sustainable behaviors. There is evidence that estimated impacts of interventions in these simple choice environments do not effectively predict outcomes in complex, real-world environments. For instance, the impact of a product-based nutrition labeling intervention tested in experimental settings was only 5% as large as the effect when implemented in real-world retail environments (38). This disparity may result from incomplete consideration of product options in complex choice environments (39).

While many health and environmental promotion approaches rely on labeling or information that is presented on product packaging, such as the use of nutrition facts panels or carbon footprint labels, an alternative approach is to provide educational information to people relevant to the products or attributes they face (40–44). Research in complex choice settings shows that educational information affects decisions even in the presence of nutrition facts panels or product-based labels (45), which results in part from changes in choice process variables—that is, people changing the sets of products and information that they consider (46, 47). However, these studies also contain hints of the effect that choice complexity may have on response to information. In a supermarket, a health message focused on fruits and vegetables led to a significant increase in healthy food purchase quantities and expenditures, but a broad message encouraging the selection of any healthy food—including fruits and vegetables—did not significantly affect purchases (48), despite the fact that all healthy products were identified by a community developed healthy food labeling system that had recently been implemented (49). Thus, simplifying the product choice setting may amplify the impact of messaging interventions.

In this study, we examine the choice of pulse-based foods in three conditions. Compared to a control condition, we estimate the impact of (1) messages about health and environmental benefits of pulses alone and (2) messages about health and environmental benefits in combination with a choice-environment intervention that allows participants to filter the complete set of dozens of products per category to view those that are pulse-containing foods, thereby reducing choice environment complexity and reducing search costs. We hypothesize that both the messaging and the messaging with filters intervention will significantly increase the likelihood of choosing pulse-based foods, but that the combined intervention will yield a significantly higher likelihood of pulse choice than the messaging alone by reducing complexity and search costs.

## 2 Materials and methods

We programmed the experiment on food choice and the subsequent survey in Qualtrics (50). The research was approved by the University of Nebraska-Lincoln Institutional Review Board (protocol #20221122409EX). To incorporate realistic levels of complexity in the food choice environment, we selected 50 food products for each of six product categories that contain pulse-based foods. We used the USDA-ARS Food Data Central Branded Foods Database to identify the prevalence of pulse-based products in categories with pulse-containing foods, and to select products to be included in the choice environment (27). The prevalence of pulse-based products is presented in Table 1. Pulse-based foods ranged from 1.4% of products in the Snacks category to a high of 7.8% of products in the Soups category.

**Table 1:** Pulse-containing and non-pulse branded food products in the six food categories.

Category	Pulse-containing foods (n)	Non-pulse foods (n)	Total foods (n)	Pulse-containing foods (%)
Frozen meals	241	3,354	3,595	6.70
Pantry staples	191	4,059	4,250	4.49
Soups	559	6,583	7,142	7.83
Snacks	513	35,214	35,727	1.44
Sauces, spreads, dips, and condiments	1,208	15,586	16,794	7.19
Frozen patties	69	1,011	1,080	6.39
Total	2,781	65,807	68,588	4.05 (weighted) 5.67 (unweighted)

Data from the USDA-ARS Food Data Central Branded Foods database; October 2022 release.

### 2.1 Product selection

While the product database featured thousands of products per category, we decided to include 50 products in each food category, which incorporates a degree of choice complexity, but represents a lower bound on most product offerings in supermarkets or online retailers. Because maintaining the average prevalence of pulse products (4%) in the database would result in only two pulse products per category, we decided to inflate the prevalence of pulse products in the experiment; this also reflects a growing interest in using pulses to create novel products (51). We selected a prevalence of 20%, resulting in 10 of the 50 products in each category being pulse products.

To populate the product set, we programmed scripts in the R programming language that searched the USDA-ARS Food Data Central Branded Foods database for pulse-containing foods within these six categories. A pulse-containing food was defined as a food that contained a pulse ingredient within its first three ingredients in the ingredient statement. A random selection of pulse-containing and non-pulse foods that appear in the database were selected to appear in the choice environment. We then gathered information about ingredients, nutrients, and product images for all pulse and non-pulse products. If we were unable to find information or a product image for a product, we replaced that product with an appropriate substitute item (e.g., replacing a pulse-based food with another pulse-based food or a non-pulse food with a non-pulse food). We collected nutrition information for each product and then adjusted nutrient values to a normalized serving size for each food product category based on FDA guidance (52). The reference serving sizes are presented in Supplementary Table S1. In the food choice task, participants viewed an image of the product. Under the image, the product name, select nutrient information (calories, saturated fat, sodium, dietary fiber, added sugar, potassium, iron, and calcium), and the per-unit product price were displayed.

### 2.2 Consumer product choice task

Participants were recruited from Prolific (53), an online survey recruitment platform, and directed to a link to our Qualtrics survey. We used the randomization feature in Qualtrics to randomly assign participants to one of three conditions. The three conditions were (1) Control, (2) Pulse Health and Sustainability Information, or (3) Pulse Health and Sustainability Information with Product Filtering. Participants in all conditions completed an informed consent process. If a potential participant was 19 years of age or older and agreed to participate in the research, they then proceeded to a brief set of instructions about the choice task. Participants also read a cheap talk script directing them to approach the choices as though they would make actual purchases—paying real money and receiving real products—even though the choices were hypothetical. Cheap talk scripts are an effective method to mitigate biases in hypothetical choices by drawing participants’ attention to the tradeoffs that the money spent on a product would entail (54).

After reading the instructions and cheap talk script, the next step of the process differed slightly among the three conditions. Participants in the *Control* condition proceeded straight to the first food category to choose among the 50 available products. Participants in the *Pulse Health and Sustainability Information* condition read a brief, simple text about the health and environmental benefits of pulses and then progressed to the first category to make a food choice. We used a simple text because this has been found to be effective across knowledge levels and to out-perform more complex texts (55, 56). The text that participants in the message condition read was: “*Choose pulses for your health and the environment! Pulses—beans, chickpeas, lentils, and dried peas—provide many benefits for human health. Plus, the production of pulses has a low impact on the environment. Choosing foods that contain pulses can improve your and your family’s health and can help protect the environment.*” Participants in the *Information and Filtering* condition read the same text as those in the *Pulse Health and Sustainability Information* condition and then had the option to view all 50 available products or to filter the full set of products to only the pulse-containing products for each of the six product categories. That is, for example, participants could choose to filter products to pulses in the Soup category but view all products in the Snack category. The participants’ choices in this step determined the set of products they viewed in each category.

In all three conditions, participants chose one product from each category or indicated that they would not choose any of the available products. Product categories were displayed in a random order to avoid order effects in product choice; however, the “None of these” option was always at the end of the list. After making decisions in all six categories, participants responded to a short survey with questions about beliefs and subjective (i.e., self-assessed) knowledge about health and sustainability benefits of pulses, satisfaction with the food choices made, and demographic characteristics, among others.

### 2.3 Data and analysis

We analyzed data using R statistical software (57). We calculated summary statistics for demographic variables for the full sample and for the three conditions. We conducted chi-square tests for differences in the distribution of variables among conditions. We also calculate the average number of pulse-foods chosen by respondents in each condition; for this calculation, individuals who indicated that they would not purchase any of the available foods were counted as not choosing a pulse food (rather than being omitted from the analysis). Next, we created a panel dataset of the foods selected, with one row for each participant’s choice in each of the six categories, resulting in six observations per participant. We used a logistic regression with cluster-robust standard errors to analyze whether a pulse-based food was chosen. The key independent variable was the condition to which a participant was randomly assigned (with the control condition being the base category). We also included variables controlling for the six different food categories (with Frozen Dinners and Entrees as the omitted category). We implemented a robustness check of the main results by incorporating demographic variables capturing the gender, age, education, and income. We report odds ratios and 95% confidence intervals for all independent variables. Data and code are available in an OSF repository.^1^

## 3 Results

We first report the summary statistics of the demographic characteristics of the full sample, as well as by condition (Table 2). The survey was completed by 1,128 individuals, with 379 respondents in the control condition, 372 in the messaging only, and 377 in the messaging and filter condition. The full sample was fairly balanced in terms of gender; 55% of participants were female. Most respondents—nearly 75%—were between 25 and 54 years of age. Over half of respondents had completed a college degree or higher. Just over 70% of respondents reported an annual household income of less than $100,000. While there was some variation in the distribution of respondents’ answers among the conditions in the experiment, there were no statistically significant differences.

**Table 2:** Demographic characteristics of the full sample and experiment conditions.

Category	Full sample	Control	Message	Message and filtering
Female (%)	55.2	54.1	56.5	55.2
Age (%)				
19–24	6.4	6.9	6.5	5.8
25–34	26.0	25.1	25.8	27.1
35–44	25.7	24.8	24.7	27.6
45–54	21.6	23.5	21.8	19.6
55–64	11.5	10.4	12.1	12.2
≥65	8.3	8.7	8.9	7.4
Prefer not to respond	0.4	0.8	0.3	0.3
Education: college or higher (%)	54.1	54.6	55.6	52.0
Income (%)				
0–20 K	9.8	9.8	10.2	9.5
20–40 K	17.6	18.5	18.0	16.4
40–60 K	19.2	21.1	17.5	19.1
60–80 K	15.0	12.7	17.5	14.9
80–100 K	11.3	10.8	12.9	10.3
100–120 K	6.7	7.1	7.0	6.1
120–140 K	5.3	6.1	4.6	5.3
140–160 K	5.1	5.3	3.2	6.9
160–180 K	2.2	1.3	2.4	2.9
180–200 K	1.5	2.4	0.8	1.3
>200 K	3.1	2.4	3.0	4.0
Prefer not to respond	2.9	2.6	3.0	3.2
N	1,128	379	372	377

Data from survey.

**Figure 1:**
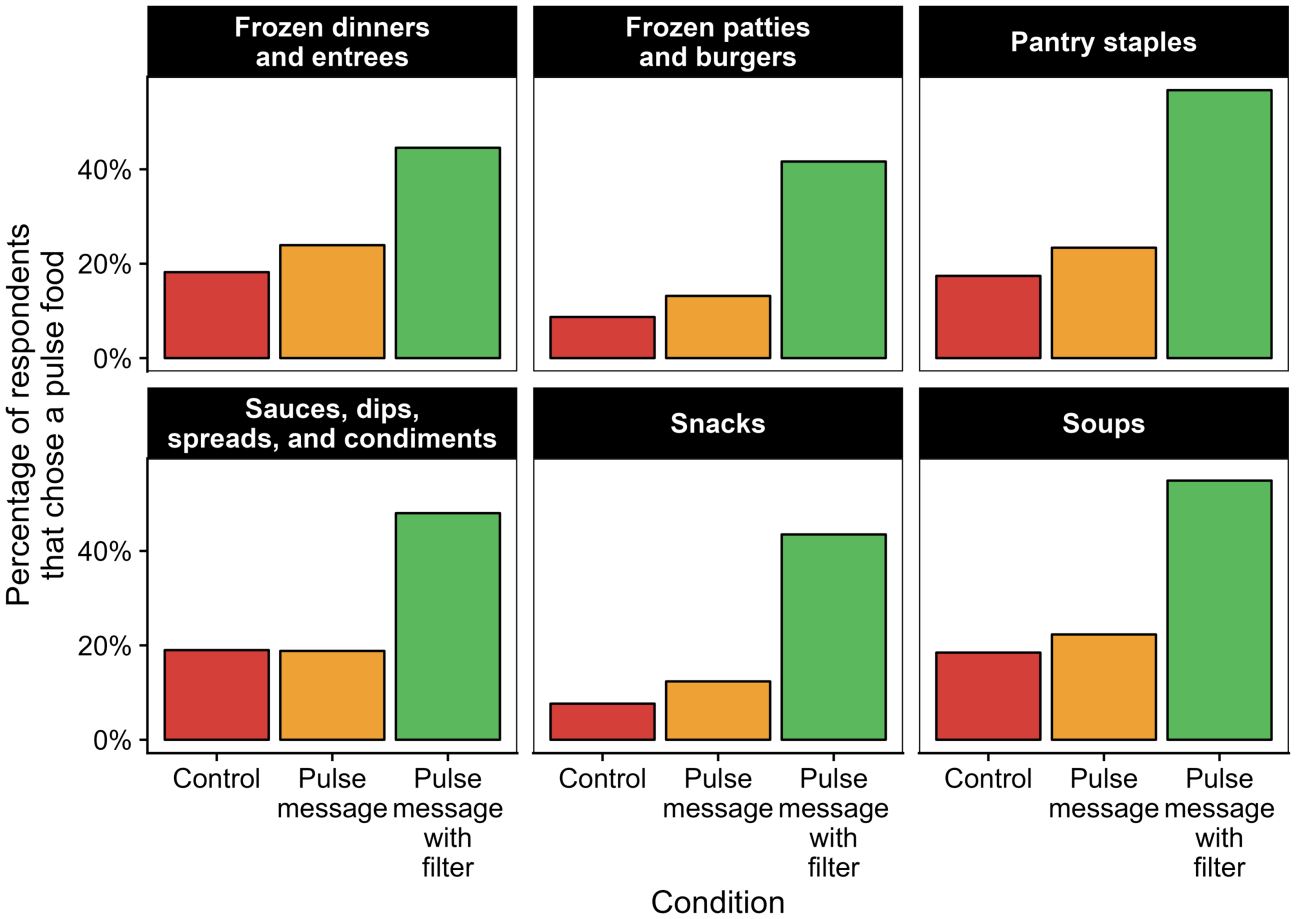
Percentage of respondents choosing a pulse food in control, pulse message, and pulse message with filter conditions.

Next, we report the mean number of pulse-based foods chosen in the experiment by participants in each condition. Given the design of the experiment, each individual could select one item from each condition, meaning that each item could either be a pulse-based food, a non-pulse-based food, or individuals could indicate that they would not select any of the available foods. For the figure and analyses below, we examined pulse-based foods as the outcome of interest and combined choices of non-pulsed based foods and responses that participants would not select any available item. Across the six food categories, participants indicated that they would not purchase any of the products for approximately 9% of choices (9.7% in the control condition, 8.4% in the messaging condition, and 9.3% in the messaging and filter condition). Figure 1 presents the percentage of participants choosing a pulse-based product in each food category.

To control for other influences on choice, we report the results of a logistic regression analysis with cluster robust standard errors of a panel dataset of participants’ choices in the experiment. We report two versions of the analysis. The first features the condition variables and control variables for each food category. In the second analysis, we add demographic variables. We report odds ratios and 95% confidence intervals in Table 3.

**Table 3:** Logistic regression of choice of pulse-based products, with individual-specific cluster robust standard errors.

	1.	2.
	Odds ratio (95% CI)	Odds ratio (95% CI)
Intercept	0.19 (0.16, 0.23)	0.07 (0.05, 0.13)
Condition (Ref: control)		
Messaging	1.34 (1.09, 1.66)	1.34 (1.09, 1.65)
Messaging + Filter	5.44 (4.45, 6.65)	5.83 (4.76, 7.14)
Product category (Ref: frozen dinners/entrees)		
FPB	0.63 (0.53, 0.75)	0.62 (0.52, 0.74)
PS	1.21 (1.03, 1.43)	1.22 (1.03, 1.45)
SDSC	0.99 (0.82, 1.18)	0.99 (0.82, 1.18)
Snacks	0.63 (0.53, 0.74)	0.62 (0.53, 0.74)
Soup	1.18 (0.99, 1.39)	1.18 (0.99, 1.41)
Female		1.15 (0.97, 1.36)
College		1.87 (1.55, 2.25)
Age (Ref: 19–24)		
25–34		1.66 (1.12, 2.45)
35–44		1.77 (1.19, 2.64)
45–54		1.43 (0.95, 2.14)
55–64		1.64 (1.04, 2.58)
≥65		1.74 (1.08, 2.81)
Prefer not to respond		1.72 (0.77, 3.80)
Income (Ref: $0–20 K)		
$20–40 K		1.16 (0.80, 1.69)
$40–60 K		1.01 (0.70, 1.45)
$60–80 K		0.93 (0.63, 1.37)
$80–100 K		0.88 (0.58, 1.33)
$100–120 K		1.12 (0.72, 1.73)
$120–140 K		0.65 (0.38, 1.12)
$140–160 K		0.81 (0.51, 1.27)
$160–180 K		0.69 (0.39, 1.22)
$180–200 K		1.36 (0.72, 2.54)
>$200 K		0.59 (0.27, 0.91)
Prefer not to respond		1.63 (0.94, 2.81)
AIC	7151.3	7051.8

Data from six product choices made by 1,128 respondents. FPB, frozen patties and burgers; PS, pantry staples; SDSC, sauces, dips, spreads, and condiments; the omitted food category is frozen dinners and entrees.

The results from the experiment show that both interventions significantly increased the likelihood that a pulse-based product was chosen. The odds that a participant who received messaging about the health and environmental benefits of pulses selected a pulse-based product were 1.34 times greater than participants in the control condition. However, the impact on the likelihood of choosing a pulse-based food product significantly increased when individuals could reduce search costs for pulse products by filtering the full product set to only include pulse-based foods. In this condition, individuals were 5.44 times more likely to select a pulse-based food than in the control condition. The estimated impact of the messaging with filter condition is also significantly greater than the messaging condition alone based on a linear hypothesis test (*p* < 0.001). Additionally, we find significant differences in the odds that individuals choose pulse-based foods across the six different food categories despite all categories having 20% of the foods in the choice set being pulse-based foods.

The addition of demographic characteristics does not weaken the relationship between experiment condition and the likelihood of a pulse-based food being chosen. The odds ratio in the messaging condition with demographic variables (Table 3, column 2) is identical to the first regression (Table 3, column 1). The odds ratio for the messaging with filter condition is slightly larger—5.83 vs. 5.44—with the addition of demographic variables. This may be due to slight and non-significant differences in the distribution of demographic characteristics across conditions. For instance, having a college degree or more education is positively related to the likelihood of selecting a pulse-based food, but the proportion of participants in the messaging with filter condition is slightly lower than in the other two conditions, which may have led to the increase in the estimated coefficient of the condition itself. As with the first model, the estimated impact of the messaging with filter condition is again significantly greater than the messaging condition alone based on a linear hypothesis test (*p* < 0.001).

Food category control variables are additionally almost identical to the first analysis. Among demographic characteristics, age and education have the most consistent significant relationship to the choice of pulse-based foods. All included age categories were significantly more likely to select pulse-based foods than the omitted 19–24 years of age category (the youngest respondents). Individuals with a college degree or higher were 1.87 times more likely to select pulse-based foods than individuals with less than a college degree. There was no significant gender-based difference in the likelihood of selecting pulse-based foods. Income categories were not systematically significant, and there was no clear pattern between increasing income and likelihood of selecting pulse-based foods. Only one income category—more than $200,000—was significantly different than the omitted (<$20,000) category. Individuals in the high-income category were less likely to choose pulse-based products.

## 4 Discussion

The complexity of real-world food retail environments may limit the effectiveness of the most widely used methods to promote healthy and/or sustainable food choices. While many experiments on the impact of label-based information find significant improvements in relevant outcomes (38, 58–61), field or natural experiments conducted in complex real-world retail settings find markedly smaller to no impact of the same labels (31, 38, 62–65). A frequent finding in studies on the impact of nutrition information or labels on food choice is that many consumers report not observing product information, and only a small fraction of those who do notice it report using it (66) or seeking it out (67). Research documenting elements of the choice process among individuals facing large product assortments finds that many shoppers consider only a small subset of available products, preventing them from comparing product-specific labeling or information (39). Many purposefully limit the number and type of products to be considered (46, 47), leading to correlations between individual characteristics, such as body weight status, and the nutritional quality of products examined during the choice process (68).

In the context of pulses, prior research found that large numbers of food items—and relatively few pulse-based food offerings—were a barrier to identification of pulse products in a virtual reality supermarket (25). Although we find a significant increase in the number of pulse-based food products chosen when participants were exposed to a message about the health and environmental benefits of pulses, our choice sets contained approximately four times more pulse-based food products proportionally than the USDA database we used to identify candidate products suggested were present among the products available in those categories (see Table 1). Providing a tool that simplified the choice environment—the ability to filter the total set of products in each product category—resulted in a significantly larger increase in the choice of pulse-based foods.

The finding that combining messaging with the ability to filter leads to significantly more pulse choices than messaging alone may reflect different impediments to the choice process. For instance, it may simply reflect the effect of a more difficult search process. Product search can lead to suboptimal choice outcomes, as well as search fatigue (69, 70).

Additionally, there could be impacts on cognitive processes during choice in complex choice environments. While taste appears to be automatically and quickly integrated into the choice process, health attributes of products are integrated more slowly, if at all, during food choice (71–74). As most health implications of food choices occur in the future, this may reflect a general tendency to asymmetrically consider current rather than future opportunity costs of options (75). Researchers have even found that people who are actively trying to lose weight lose track of dieting goals in the face of preferred foods (76). However, health-oriented prompt messages seem to successfully redirect attention to health (77), even among non-dieters (71).

The effectiveness of messages themselves may be influenced by the complexity of the environment. In a study on goal-oriented healthy food prompt messages in a rural supermarket, shoppers were exposed to one of three conditions: a control, a message narrowly focused on fruits and vegetables, or a message broadly focused on any healthy foods (48), identified by a locally designed healthy food labeling system (49). While healthy food purchases significantly increased in the condition narrowly focused on fruits and vegetables, the broadly focused message did not yield a significant change in purchases relative to the control condition. This result occurred even though fruits and vegetables constituted a subset of the relevant products in the broad condition, meaning that shoppers in that condition should have found at least as many healthy products that they wanted to purchase as shoppers in the message condition narrowly focused on fruits and vegetables.

Stimulus-rich settings, such as food retail environments, may present multiple barriers to the purchase of healthy and/or environmentally friendly foods. Complex choice environments may reduce shoppers’ willingness to search for a product with a preferred set of attributes. On the other hand, complex environments may distract from other long-term goals of consumers, such as making healthy or environmentally friendly choices. Unless individuals systematically consider the health and/or environmental impacts of the products they are facing, consideration of attributes that promote environment sustainability may be forgotten or overlooked during the process of shopping. While research shows that priming consideration of sustainability increases the likelihood that consumers choose more environmentally friendly products (78), this research has not been conducted in complex choice settings, which are more cognitively demanding to navigate and reduce the number of attributes and/or products that consumers consider when making choices (39, 79). However, research on health-focused messaging in complex brick-and-mortar and online environments suggests that these interventions can work (45, 80), although careful thought has to be given to the design of the intervention (48).

While we focus on the impact of messaging and a paired messaging and filter intervention on the choice of pulse-based foods in a complex choice environment, there are other consumer-specific variables that would provide insight on the low levels of pulse consumption in the US and should be studied in future research. For instance, lack of knowledge, incorrect beliefs, and constraints on cognitive resources—such as attention and memory—likely all contribute to the low levels of pulse consumption in the US by limiting consideration of pulses in decision-making (81–85). Because pulses have favorable nutrient and sustainability profiles, consumers should find pulses to be attractive food options. However, since many health benefits of pulses are not widely known (23), people are less likely to seek them out.

Our research has some limitations worth noting. First, product choices were hypothetical. While hypothetical bias is a concern of all research featuring hypothetical choices, we followed an established method—the use of a cheap talk script—that has been widely tested and found to ameliorate the impact of hypothetical bias (54). A short-term goal is to repeat the research using real, binding choices in order to test the replicability of these findings when individuals are making non-hypothetical choices.

Additionally, there are a few design choices that were built into this study that we plan to explicitly test in ongoing work. First, we plan to examine the impact of the prevalence of pulse-based food products within food categories. As noted earlier, we increased the percentage of pulse-based products in the food categories participants faced in this research to 20% of the products offered from the approximately 5% found in the USDA FoodData Central list (27). We made this choice to provide more than two or three pulse foods per category, which is what would result from 5% of a 50-item product set being pulse-based products. In future research, we will vary the prevalence of pulse foods to examine the impact of pulse food availability within the food categories. Next, while the aim of this research project was to examine how providing a tool that allowed respondents to deal with choice complexity—the presence of large numbers of products—to easily find pulse products affected the impact of a message on the health and environmental benefits of pulses on food choices, it would be useful to compare choices in a condition in which participants could filter options but were not exposed to messaging. We will investigate this in an upcoming research project.

Our findings suggest that messaging combined with features that allow shoppers to simplify the choice environment may have a markedly larger effect in promoting the selection of key attributes related to nutritional quality and environmental impact outcomes during food choice than messaging alone. The interaction between the decision environment and cognitive processes is an important factor that should be considered when designing interventions to ensure that choice complexity is accounted for and, when possible, mitigated to prevent a diminution of the effectiveness of the intervention.

## Data Availability

The datasets presented in this study can be found in online repositories. The names of the repository/repositories and accession number(s) can be found at: https://osf.io/8kfn2/?view_only=29ed131f56884595a0de9cd559d87a73.

## Footnotes

^1^ https://osf.io/8kfn2/?view_only=29ed131f56884595a0de9cd559d87a73
