# Supplementary material for: The impact of health and environmental messaging with and without product filtering in complex retail markets: the case of pulses
Source: Front Nutr. 2024 Sep 25;11:1454271. doi: 10.3389/fnut.2024.1454271 (PMC11462336; doi:10.3389/fnut.2024.1454271)
Supplement: Supplementary file 1 [file Table_1.DOCX]

Supplementary Materials Table 1: Standardization of serving sizes for calculation of nutrient information.

| Pantry staples | ≤150g serving size standardized to 50g |
| --- | --- |
|  | >150g serving size standardized to 225g |
| Snacks | Standardized to 28g |
| Soups | ≤180g serving sizes standardized to 120g |
|  | >180g serving sizes standardized to 240g |
| Frozen Dinners and Entrees | Standardized to 250g |
| Frozen Patties and Burgers | Standardized to 120g |
| Sauces/Spreads/Dips/Condiments | ≤20g serving sizes standardized to 12g |
|  | >20g serving sizes standardized to 28g |
